# Advance care planning conversations with palliative patients: looking through the GP’s eyes

**DOI:** 10.1186/s12875-018-0868-5

**Published:** 2018-11-28

**Authors:** Anne B. Wichmann, Hanna van Dam, Bregje Thoonsen, Theo A. Boer, Yvonne Engels, A. Stef Groenewoud

**Affiliations:** 1 Radboud Institute for Health Sciences, IQ healthcare, Radboud university medical center, Nijmegen, The Netherlands; 2 Department of Anesthesiology, Pain and Palliative Medicine, Radboud university medical center, Nijmegen, The Netherlands; 3 Section Ethics, University Kampen, Kampen, The Netherlands

**Keywords:** Advance care planning, Palliative medicine, General practice, Qualitative research

## Abstract

**Background:**

Although it is often recommended that general practitioners (GPs) initiate advance care planning (ACP), little is known about their experiences with ACP. This study aimed to identify GP experiences when conducting ACP conversations with palliative patients, and what factors influence these experiences.

**Methods:**

Dutch GPs (*N* = 17) who had participated in a training on timely ACP were interviewed. Data from these interviews were analysed using direct content analysis.

**Results:**

Four themes were identified: ACP and society, the GP’s perceived role in ACP, initiating ACP and tailor-made ACP. ACP was regarded as a ‘hot topic’. At the same time, a tendency towards a society in which death is not a natural part of life was recognized, making it difficult to start ACP discussions. Interviewees perceived having ACP discussions as a typical GP task. They found initiating and timing ACP easier with proactive patients, e.g. who are anxious of losing capacity, and much more challenging when it concerned patients with COPD or heart failure. Patients still being treated in hospital posed another difficulty, because they often times are not open to discussion. Furthermore, interviewees emphasized that taking into account changing wishes and the fact that not everything can be anticipated, is of the utmost importance. Moreover, when patients are not open to ACP, at a certain point it should be granted that choosing not to know, for example about where things are going or what possible ways of care planning might be, is also a form of autonomy.

**Conclusions:**

ACP currently is a hot topic, which has favourable as well as unfavourable effects. As GPs experience difficulties in initiating ACP if patients are being treated in the hospital, future research could focus on a multidisciplinary ACP approach and the role of medical specialists in ACP. Furthermore, when starting ACP with palliative patients, we recommend starting with current issues. In doing so, a start can be made with future issues kept in view. Although the tension between ACP’s focus on the patient’s direction and the right not to know can be difficult, ACP has to be tailored to each individual patient.

**Electronic supplementary material:**

The online version of this article (10.1186/s12875-018-0868-5) contains supplementary material, which is available to authorized users.

## Background

Advance care planning (ACP) is the ongoing process in which a patient, his or her family and healthcare provider reflect on the patient’s goals, values and beliefs, and define goals and preferences for future medical treatment and care, ideally following an exploration of the patient and caregiver’s knowledge, fears, hopes and needs [1, 2]. In addition to being an important means in extending patient autonomy, early initiation of ACP in palliative patients has been shown to improve patient and family satisfaction and quality of life (QoL) [3, 4], improve concordance between preferences for care and delivered care [5] and to be positively correlated with less aggressive care (such as ICU visits) [4, 6].

Despite these benefits, ACP is hardly undertaken in palliative patients in current practice [7–9]. Organisation and delivery of healthcare is predominantly reactive, and discussions about wishes and needs for future care still form only a small part of daily practice [10–12]. Poor knowledge of patient preferences might result in care being offered in a way they might not otherwise have chosen [3, 13]. This might harm QoL by preventing patients from leading meaningful lives and preparing for death [14]. This makes timely discussion of end-of-life (EoL) issues ever more important for patients, relatives and healthcare professional as well as society more broadly.

It is often recommended that the patient’s general practitioner (GP) should be the initiator of ACP [15, 16]. Continuity of care in general practice creates an opportunity for a longstanding doctor–patient relationship [17]. Often, the GP has already known the patient and his or her social context for a considerable length of time. Besides, since patients often see several medical professionals, the GP ideally is the linchpin between these several disciplines and the patient. Despite the fact that ACP is becoming increasingly important, the considerable body of knowledge concerning the value of ACP and the multiple ACP training programmes that have been developed during the past few years [18–20], little is known about how GPs envisage and experience their role in ACP. Besides, previous studies mostly focused on the organisation and coordination of palliative care [21, 22] and on specific patient groups [23, 24]. The aim of this study was to identify GPs’ experiences when conducting ACP conversations with palliative patients, and what factors influence these experiences.

## Methods

### Study design

A qualitative research design was chosen in order to obtain in-depth insights into GP experiences with timely ACP discussions with palliative patients. Our study comprised semi-structured interviews with GPs located at different practices throughout the Netherlands. Data were collected from May to December 2016.

### Participants and data collection

We recruited GPs who had participated in an ACP training programme recently developed by the Dutch College of General Practitioners (*Nederlands Huisartsen Genootschap*; NHG). This training consisted of two modules with a total duration of six hours. It comprised theory and peer-review practice and focused primarily on discussing EoL issues (Additional file 1). Purposive sampling of trained GPs was employed to ensure the sample represented a diverse range of GP and practice characteristics such as age, sex, experience and size and location of the practice (Table 1). Non-participants mostly declined because of time issues. Recruitment was stopped after saturation was reached and confirmed in three additional interviews.

**Table 1:** Characteristics interviewees

Code	Gender	Age range	Years experience	Other relevant information
R1	Female	50–55	20 yrs.	
R2	Female	50–55	15 yrs.	Lot experience with elderly
R3	Male	30–35	5 yrs.	Acting GP
R4	Male	55–60	29 yrs.	GP in practice with lot of elderly
R5	Male	50–55	25 yrs.	Finished staff college palliative care
R6	Male	60–65	34 yrs.	Lot of experience with palliative care
R7	Female	50–55	18 yrs.	Finished a palliative care course
R8	Male	35–40	7 yrs.	
R9	Female	40–45	7,5 yrs.	
R10	Female	35–40	5 yrs.	
R11	Female	50–55	15 yrs. GP.	
R12	Male	55–60	25 yrs.	Affiliated with PATZ group (palliative care at home)
R13	Male	55–60	25 yrs.	
R14	Female	40–45	15 yrs.	Teacher in GP education
R15	Female	40–45	15 yrs.	
R16	Female	50–55	22 yrs.	Runs big solo-practice
R17	Female	35–40	3,5 yrs.	Acting GP

An interview guide was developed starting from the research gap identified in the introduction, and it was inspired by the framework by Boer et al. [25] The guide was discussed and amended if needed by ABW, SG, HvD and TB. It consisted of four topics, each comprising multiple open questions (Additional file 2). Nine interviews were conducted by two authors (ABW, HVD and/or SG) and the remaining eight by one author (ABW or HVD). See Table 2 for more information regarding researchers’ characteristics. At the start of each interview, participants were informed that the goal was not to evaluate the training itself, but to talk about their experiences with ACP with palliative patients.

**Table 2:** Characteristics interviewers

Code	Initials	Gender	Age	Occupation and experience
I1	A.B.W.	Female	30	PhD candidate, master degrees in Health Sciences and Philosophy. 3 years research experience on multiple EoL projects, experienced in conducting interviews (previously held ±30 interviews), no experience in patient care.
I2	H.v.D.	Female	24	Master student Medical Sciences. Five months experience as a researcher, limited experience in conducting interviews (previously held ±10 interviews), limited experience in patient care.
I3	S.G.	Male	40	PhD with PhD degree in Health Sciences and master degree in Philosophy. 10 years research experience on multiple EoL projects, experienced in conducting interviews (previously held ±50 interviews), no experience in patient care.

The first few interviews took place face-to-face at a location most convenient for the interviewee, so that the interviewers could get a good feeling with the matters discussed. Subsequently, interviewees were given the choice between a face-to-face or a telephone interview. The latter was chosen in all cases for geographical or other reasons making the interview more comfortable for interviewees. The interviews were conducted in Dutch and lasted from one to one-and-a-half hour and were recorded, for which all participants gave consent. The interviews were transcribed verbatim by an official agency, and names of participants were anonymised.

### Data analysis

Since the goal was to *understand* the experiences and motivations of GPs, an interpretative approach was used, in which the focus is understanding the world as others experience it [26, 27]. A constant comparative method was chosen for data analysis the data [27, 28]. First, two authors (ABW and HVD) independently read, reread and openly coded the first seven transcripts. These codes were discussed and compared until an initial codebook was established. Two researchers (ABW and HVD) independently analysed the rest of the interviews by applying the coding framework and modifying it through an inductive, iterative process. In this phase, codes were discussed, added, modified or merged if necessary. Finally, these codes were discussed, axially coded and combined into categories and themes (by ABW, SG and YE). The software programme Atlas.ti 7 was used in managing and analysing the data. In reporting the data the Standards for Reporting Qualitative Research was used [29].

## Results

Seventeen of 25 invited GPs participated; see Table 1 for demographics of participants. Two interviews were conducted face-to-face and fifteen by phone. Saturation was reached after fourteen interviews.

After having analysed all transcripts, four themes were identified in the fifty-three codes and nine categories. These themes concerned bundles of GP experiences about ACP and society, GPs’ perceived role in ACP, initiating ACP and tailor-made ACP.

### Theme i. Acp and society

Interviewees emphasised ACP is a ‘hot topic’, and mentioned that because of this trend, they are more primed to discuss EoL issues in practice. *‘Anticipating “How to deal with it? What will we know in the future?” That is becoming a trend. It is a hot item also in the literature. Which is stimulating.’* (R4) Interviewees also recognised a more proactive attitude in their patients, for example, when people are anxious about developing dementia or otherwise losing capacity. And although these patients according to interviewees sometimes have false expectations regarding ACP and the GP’s role in it, as if it were a matter of checking items off a list, it opens doors to discussion.

Because of this, ACP was regarded a welcome development. At the same time, interviewees mentioned that the current tendency towards a ‘malleable’ society leads to death not being a normal part of life anymore, making it difficult for them to start ACP discussions: *‘In my opinion, death nowadays is not experienced as being part of your life anymore (…) People find it hard to talk about it. Which makes it difficult for me as GP [to start ACP, red].’* (R1).

The same interviewee suggested governments could play a role in normalising communication about death and dying, *“for example through television spots. Along the lines of ‘you organised your house, your holiday, etcetera. Have you also considered how you would like to organise the last years of your life, or discussed it with your GP?’”* Other GPs subsequently warned of a possible negative image of ACP, for example arising out of feelings of negligence. *“What is the government’s objective? Are they concerned I’m getting too old? Am I too expensive?”* (R12) Proactively mentioning the approaching death might be experienced as an efficiency measure for patients as well as for the general public. This may lead to a negative social perception of ACP.

### Theme ii. The GP’S perceived role in ACP

#### Fitting the profession

Interviewees envisaged an important role for themselves in ACP. Conducting ACP was perceived as a typical GP task because of the longstanding relationship between the GP and the patient and his or her social context, and the GP’s easy accessibility. *‘We have been doing this for so many years now. We have known a lot of our patients for a long period of time. We can more or less predict the attitude of a certain patient.’* (R6) However, it was also argued that having ACP conversations with palliative patients should be better integrated into GP education.

Furthermore, a distinction was made between the GPs ‘medical’ and ‘social’ role. With regard to the medical role, it was mentioned that administration of medication should be well adjusted and that a ‘do not resuscitate’ directive should be in place. However, most GPs saw their social or multidimensional role as being at least as important. They felt this role is inseparably linked to the palliative phase. One interviewee described this role as helping people ‘wrap up’ their lives: *‘Ideally…you want one to be able to quietly finish her or his life (…) to be ready for it, as it were…’* (R5) Numerous interviewees felt that this multidimensional approach made ACP a rewarding component of their work: *‘That’s when you think: “Why? Then everyone will die.” But you can get very close to “What is really important to you? What can I do for you?”’* (R1).

At the same time, it was indicated that it can feel safer to revert to the ‘medical-technical’ role, as it makes it easier to avoid any emotional confrontations between the patient and the GP. Because this ‘*…is less the case, yes [emotions playing a role in a standard conversation, red.]. In that case it usually turns out to be a more technical story, doesn’t it?’* (R3) Another interviewee explained he sometimes has to suppress his emotions so that the situation doesn’t get too close. *“Sometimes you have deathbeds of which you think ‘I’m rather getting swept up in it too much..’ People confide in you só much, you’re so intimate, that I sometimes feel it is a little too much for me’.”* (R12).

Some GPs felt the need for ACP guidelines. Most of them however, considered EoL care too personal to capture in guidelines because *‘It is your view on how you want it, it is sort of a personal touch you give to your job. I don’t think you can put in a protocol: this must be part of it, or else you did not do a proper job.’* (R4).

#### Time: Needed but scarce

Two issues regarding time were discussed. On the one hand, GPs emphasised that time is needed, because *‘If you are too busy yourself, how could you be able to listen? In that case you could ask your questions, but how could you be able to really listen to the patient? Or do something through unspoken communication?’* (R7) Interviewees noted that GPs, more than medical specialists, have time. On the other hand, lack of time was considered a barrier to starting conversations due to increasingly full agendas. *‘You’re running late, and still have to do two visits. While sitting there, you’re thinking: if I ask this question now, where would it get me? (…) Time, planning…those are real barriers to asking these kinds of questions.’* (R12).

Thinking ahead was proposed as a solution to these issues: *‘With someone of advanced age, and who’s condition is deteriorating, who I have to visit for a tickling cough, that I mention it briefly then (…) What is most important is that you follow up on it.’* (R7) When doing that, it was argued, conversations can be more easily planned and held during regular working hours. Another advantage of thinking ahead is that *‘if you feel that there is resistance [in talking about the EoL, red.], it is better you know it up front instead of in the last week of life.’* (R12).

### Theme iii. Initiating ACP

The third theme regards the initiation of ACP. Categories within this theme concern starting the conversation and issues in the relation with the hospital in initiating ACP.

#### Starting the conversation

Allowing patients to settle their wishes and needs for future care, and enabling them to prepare themselves for what might be coming were mentioned as reasons for starting ACP. *‘Having these conversations is a matter of great urgency. Not because you want to outline all possible scenarios, but because you know what kind of scenarios patients might expect’* (R12), one of the GPs stressed. Interviewees mentioned that because of this urgency, they try to time ACP more proactively nowadays. *‘I used to be more reactive; now I try to take a more proactive stand…Before, I would say: “no, you’re not doing too well, but we will make the best of it.” Now, I would more likely say: “Well, try to think about it yourself, how do you envisage it?”’* (R4).

Nevertheless, interviewees mentioned that although it is not ideal, they often only start ACP discussions when the patient can no longer be curatively treated, when the disease has reached a critical stage. *‘It is not ideal to ask about their wishes when something happens, i.e. when someone must be hospitalised. But usually however, that is the case.’* (R2) Starting ACP earlier can be hard because proactively starting ACP can be difficult and even do harm, interviewees felt. Therefore, ACP is often initiated late, also because patients might still hope for a cure before that stage. *‘If you ask about it at a too early stage, the patient looks at you with wide eyes, thinking you have already decided when he will die (…) At that moment the patient is not yet ready for it emotionally. In that case it comes as a surprise, and that might incite annoying reactions.*’ (R6).

Interviewees found it easier to start ACP with patients with cancer than with other patients as, they observed, a relatively strict separation can be made between the curative and palliative phase in their disease trajectories. Initiating ACP was perceived to be easier with proactive patients who initiate conversations themselves, or are open to having them. For example, with the previously mentioned patients who are anxious about dementia or want to have everything ‘settled’, and thus actively approach their GP. “*When they present their will, having downloaded a lot of information, thinking this is it, I have done what I had to do (…) that’s not how it works, of course. But it is a nice occasion to start discussing these matters”*’ (R4).

#### When ACP is difficult

As opposed to patients with cancer, interviewees felt that timing and starting ACP with chronically ill patients is hard, since their illness trajectory is less predictable. *‘I mean, why would it [in patients with heart failure or COPD, red.] suddenly be over? And when it’s over, it will be a slow process (...) When is the right time to bring it up? That is rather difficult.’* (R12) The trajectory also influences patients’ attitude and goals. According to interviewees, chronically ill patients are often focused on recovery instead of their general decline, for example after having experienced an exacerbation. *‘Most of the patients with COPD I saw on the way to the end held on to their lives very tightly (…) The conversations I had with these patients were mainly about “how can I stay alive as long as possible”.’* (R13) It was stressed that with chronically ill patients, starting with current issues and clear milestones makes opening the discussion more fruitful, *“especially with patients with COPD it’s difficult to bring it up, because they always feel a little better after treatment. The same goes for heart failure. After treatment, it’s ‘business as usual’. And after an exacerbation, you get a little further.”* (R5).

Another difficulty interviewees experienced in timely initiation of ACP discussions was when their own ideas about ACP did not correspond with those of their patients. For example, when the GP’s belief that it is good to discuss the EoL and associated emotions is not being answered: *“I once had a patient who stopped visiting me because I had tried to talk about the EoL.. Finally he did want a dormicum pump so I had to put that in place.. that left me feeling I was the executioner.”* (R3) Interviewees mentioned it is easier if the patient’s view and choices could be your own, *“for example a woman of my age, who more or less lived the same way I do.. The fact you can level makes a difference, I think.”* (R1) GPs agreed that in any case, they should continue raising the issue, making clear they are open to discussion. At the same time it was mentioned that whatever reason patients have for not wanting to engage in ACP, GPs should respect this. Because *‘choosing not wanting to know is also a form of autonomy.’* (R3) An interviewee mentioned that letting the GP decide can also serve as a coping strategy for palliative patients. On the whole, interviewees agreed that ACP should not be imposed on patients because *‘When you notice that it is so frightening for a patient, or so unimaginably overwhelming, I will not force him to swallow it (…) She [a female patient, red] did sense it, she only didn’t want to explicitly and extensively discuss it all. I think it’s a big drawback these days that everything has to be made explicit. It is in the media, it’s everywhere. There’s also an indirect way to, yeah…’* (R4).

#### ACP when hospital treatment is ongoing

Another perceived barrier in timely initiation of ACP concerned patients who are still being treated in hospital. According to interviewees, they are often not open to ACP discussions: *‘I then try it [starting ACP conversations, red.], but it doesn’t work because people still have hope that they can be treated curatively. At such a moment, I think to myself “well, well, what are you starting…please don’t!” (…) But…who am I to tell them not to do it?’* (R5) Some felt their specialist colleagues do not sufficiently prepare palliative patients and that this affects the opportunities GPs have in ACP. Because *‘if people are still engaged in curative care, a lot of energy, hope and focus is aimed at getting through the treatment (…) I wouldn’t say they [curative treatment and ACP, red.] exclude each other, but there’s just very little room when someone is in the midst of curative treatment.’* (R14).

Interviewees indicated that the collaboration and communication between GPs and the hospital is missing, *“the oncology nurse doesn’t reciprocate enough. Sometimes it is not even clear who wrote you the letter, the nurse or the medical specialist.”* (R12) However, more multidisciplinary communication would be very helpful as both can use their specific knowledge in making the best plan for the patient: *‘They [medical specialists, red.] are better informed about further possibilities and I see what happens at home (…) Very often a patient is discussed in the multidisciplinary consultation in the hospital. Maybe they could also consult the GP more often: these are our plans, do they seem possible to you, too?’* (R11).

#### Responsibility costs

Within the discussion regarding GPs’ relations with hospitals, another category arose concerning the GPs’ responsibility for patients who are possibly being curatively overtreated. GPs emphasised that a lack of ACP potentially results in overtreatment in the palliative phase. And this not only has associated financial costs but, no less important, also costs in terms of QoL. Because of both costs, GPs mentioned that continuation of treatment with a curative approach in palliative patients should always be discussed. *‘ACP starts by looking at quality of life. That brings about questions with respect to efficiency. Not the other way around. With everything we do, we have to keep in mind: what do we expect from this treatment, and will that really be the result?’* (R2).

GPs mainly felt a responsibility in keeping costs low if patients have to ‘pay’ in terms of QoL, but less in controlling literal financial costs. ‘*I think quality of life is more important than the question: does it carry more costs? But it is, of course, a pity to give someone a dreary number of last months while also spending a lot of money on him. In that case it would be better to stop a few months earlier. That would save money too.’* (R12).

### Theme iv. Tailor made ACP

Although fairly obvious, GPs emphasised it is very important that ACP is tailored to each individual patient. After all, just as every person has their own lifestyle, everyone also has his or her own ‘dying-style’. *‘Everyone does it his own way. If someone fights until the end, sure there’s probably a reason for that too… That’s just how he is.’* (R5) In this context it was also mentioned that being explicit, e.g. about future wishes and needs, and preparing for what might be coming simply might not fit one’s style. *‘Some have no desire at all to discuss EoL issues. Why should you then bring up all kinds of issues, if that doesn’t fit his or her personality at all?’* (R12).

#### Changing wishes

Interviewed GPs also mentioned that in ACP it is crucial to take changing wishes into account. One of the interviewees humorously illustrated the fact that you cannot anticipate everything: *‘It is the same as having children: you have very specific ideas about that, about delivering at home, and breast feeding and all that. You would not be the first to say, I’ll deliver comfortably at home and in the bath and that by the time you have contractions the nurse says, well let’s step into a nice bath, and you say a bath?! I don’t want to step into my bath. No way. I want to go to the hospital, now!’* (R1).

When the above is taken into account, ACP can have very positive effects: *‘It brings peace and tranquillity. Also, I think, people will reflect on their situation more… they do not leave everything to the last minute, they arrange things in advance, talk about it, are able to say farewell.’* (R5).

## Discussion

In this interview study, four themes regarding GPs’ experiences when conducting ACP conversations with palliative patients and factors influencing these experiences were identified. ACP was regarded a ‘hot topic’, which was seen as a stimulator (GPs are more primed, patients are more proactive) as well as a barrier (death is not regarded a natural part of life anymore) for timely initiation of ACP. Interviewees felt they have an important role in ACP. Initiating and timing ACP was experienced as easier with proactive patients who initiate discussions themselves or are open to it, for example because of a fear of losing capacity, and when the course of disease is more predictable, which is often the case with cancer. On the other hand, they perceived conducting ACP as much more challenging when it concerned patients with COPD or heart failure. Moreover, GPs experienced difficulties when patients were still being treated in hospital, as those patients are generally not open to ACP discussions. Furthermore, it was emphasised that although this is difficult, as wishes change and not everything can be anticipated, ACP has to be tailor-made. For some patients however, discussing wishes and needs is simply not their cup of tea.

### Comparison with existing literature

An interesting mix of ACP being a hot topic and death not being regarded as a natural part of life these days was found. Just as interviewees in this study, the Economist Intelligence Unit called for encouraging people to openly discuss their EoL wishes and normalise the conversation about it [30]. Moreover, it is often recommended that GPs act as facilitators in ACP [31], and multiple ACP trainings have been developed specifically for GPs [18–20]. A survey among 516 GPs for example found that 97% felt that general practice plays a key role in the delivery of care to people approaching the EoL [32], and the majority of patients appreciate it if their GP takes the initiative [33]. Although a 2013 review found evidence for a GP attitude that patients should initiate discussions [31], our study shows interviewees feel they have a role in addressing ACP discussions, even if patients do not have an active stance towards these discussions. Like others, we found that GPs find it hard to identify and discuss palliative care needs with patients with organ failure [23, 34]. Moreover, our finding that initiating ACP is difficult when patients are still being treated in hospital is also confirmed in other literature [35–37]. It was mentioned in our study that *‘it is not ideal to ask about their wishes when something happens, i.e. when someone must be hospitalised.’* However, these clear milestones offer the possibility to initiate ACP. Research showed that these events, such as hospital admissions or exacerbations, can serve as a helpful starting point for discussions about wishes and needs [38], and may result in ACP taking place more frequently [39]. Lastly, a tension between ACP’s focus on the patients’ own direction and an in medical ethics recognized right not to know is present. Getting patients to face their approaching death is, in a way, a form of medical paternalism. Such paternalism can be justified as long as GPs acknowledge that not wanting to know (where things might be going or what possible ways of care planning are), can also be a form of autonomy. Nevertheless, this can be quite difficult for GPs, as ACP is mainly conducted in the context of anticipated deterioration. I.e., if a patient loses capacity, GPs should make use of information obtained from the ACP process to guide their decision making [40]. The concept of genuine consent, in which provision of information is cyclically adjusted to current wishes and abilities of patients, might better fit the context [41].

### Strengths and limitations

A strength of this study is that interviewees had already looked into the matter closely, as they had taken part in the ACP training of the NHG. At the same time, participants had been sufficiently interested in ACP to pursue ACP training, which might have biased the data. Also, the know-how gained from the training might have influenced their experience with actual ACP discussions. However, although generalisability of qualitative research is always an issue, this study reflects similar findings in other studies. Furthermore, although conducting interviews by telephone has been considered inferior to face-to-face interviews [42], recent research showed that rich narrative data collection can be achieved using this method [43]. Telephone interviews may even allow respondents to feel more relaxed and able to disclose sensitive information [42]. We have paid proper attention to the special character of interviews on the phone, but found no indications that in those interviews any aspects would we paid less attention than needed. Moreover, although it was repeatedly emphasised that the palliative stage is *broader* than the terminal phase, interviewees predominantly spoke about patients in their terminal phase. Despite their training, multiple GPs experienced a barrier in starting ACP conversations, the cause of which might be their focus on the terminal phase. The fact interviewees oftentimes regarded palliative patients as being terminal underlines the awareness needed for what palliative care entails.

### Implications for future research and clinical practice

A focus on the terminal phase and death might result in difficulties with starting ACP, and in ACP conversations taking place too infrequently. The goal of ACP is to define, discuss and review goals and preferences for future medical treatment and care, in order to improve the quality of the remainder of patients’ lives [1]. Death and EoL arrangements are in most cases only a small part of remaining life. Interviewees indicated that, mainly with chronically ill patients, clear milestones like exacerbations or hospitalisations can be used to start ACP. This finding dovetails beautifully with, when initiating ACP, staying close to the *now* is helpful: asking what is important for patients at *this* moment might facilitate conversations about needs and wishes in an accessible way. Useful example questions are described in the Supportive and Palliative Care Indicators Tool [44], as well as by the Mount Vernon Cancer Network. For example: ‘*What in particular are you concerned with at this moment?’* and ‘*What or who gave you support in previous situations*?’. These questions can serve as a good basis for timely initiation of ACP.

We recommend taking the above into account in future ACP training programmes, as it will lead to open and transparent conversations about realistic expectations, and subsequently wishes and needs can be tailored accordingly. Patients can benefit from this: research shows that the decline of QoL in palliative patients with false hope was larger than the decline in patients with realistic hope [45–47]. Regarding the lack of collaboration between GPs and medical specialists, which might result in overtreatment and associated costs in terms of patients’ QoL as well as societal costs, interviewees suggested regular multidisciplinary meetings or simply picking up the phone more often could help. Recent research indicated that integrated care and multidisciplinary training in ACP might bridge gaps [48, 49]. Another suggestion is better data sharing: an English initiative enabling ACP and improving communication and coordination for example, had successful outcomes (such as 77.8% of patients dying in their preferred place) [50]. Finally, research showed transitional care pathways can be effective in reducing hospital readmissions [51, 52]. We suggest future research to further look into finding ways to achieve a multidisciplinary approach in ACP, and the role of medical specialists in ACP.

## Conclusions

The fact that ACP currently is a hot topic, has favourable as well as unfavourable effects. GPs and patients are more proactive, while at the same time death is not regarded a natural part of life these days. It was suggested that normalisation of EoL conversations should be promoted. Interviewees on the whole agreed that GPs have an important role in ACP. Remarkably, GPs experienced difficulties with initiating ACP when patients are being treated in the hospital. If GPs and medical specialists would work more closely together, this could help GPs in fulfilling their role in the timely initiation of ACP. Lack of this form of collaboration might prove disadvantageous. Future research could focus on a multidisciplinary approach, and the role of medical specialists in ACP. We recommend commencing with current issues, viz. staying close to the now. In doing so, a start can be made in addressing issues that may become important in the near future. Also, the tension between ACP’s focus on the patients’ own direction and an in medical ethics recognized right not to know can be difficult. Lastly, although taking into account changing wishes and the fact that you cannot anticipate everything can be difficult, ACP has to be tailor made to each individual patient.

## Additional files

Additional file 1: Content ACP training (NHG) [53–56]. (DOCX 18 kb)

Additional file 2: Interview guide. (DOCX 16 kb)

## Abbreviations

ACP: Advance care planning
COPD: Chronic obstructive pulmonary disease
EoL: End of life
GP: General practitioner
ICU: Intensive care unit
NHG: *Nederlands Huisartsen Genootschap* (Dutch College of General Practitioners)
QoL: Quality of life

## References

[1] Rietjens Judith A C, Sudore Rebecca L, Connolly Michael, van Delden Johannes J, Drickamer Margaret A, Droger Mirjam, van der Heide Agnes, Heyland Daren K, Houttekier Dirk, Janssen Daisy J A, Orsi Luciano, Payne Sheila, Seymour Jane, Jox Ralf J, Korfage Ida J (2017). Definition and recommendations for advance care planning: an international consensus supported by the European Association for Palliative Care. The Lancet Oncology.

[2] Advance care planning and advance directives [https://www.uptodate.com/contents/advance-care-planning-and-advance-directives].

[3] Detering KM, Hancock AD, Reade MC, Silvester W (2010). The impact of advance care planning on end of life care in elderly patients: randomised controlled trial. BMJ.

[4] Brinkman-Stoppelenburg A, Rietjens JA, van der Heide A (2014). The effects of advance care planning on end-of-life care: a systematic review. Palliat Med.

[5] Houben CH, Spruit MA, Groenen MT, Wouters EF, Janssen DJ (2014). Efficacy of advance care planning: a systematic review and meta-analysis. J Am Med Dir Assoc.

[6] Khandelwal N, Kross EK, Engelberg RA, Coe NB, Long AC, Curtis JR (2015). Estimating the effect of palliative care interventions and advance care planning on ICU utilization: a systematic review. Crit Care Med.

[7] Howard M, Bonham AJ, Heyland DK, Sudore R, Fassbender K, Robinson CA, McKenzie M, Elston D, You JJ (2016). Measuring engagement in advance care planning: a cross-sectional multicentre feasibility study. BMJ Open.

[8] Heyland DK, Barwich D, Pichora D, Dodek P, Lamontagne F, You JJ, Tayler C, Porterfield P, Sinuff T, Simon J (2013). Failure to engage hospitalized elderly patients and their families in advance care planning. JAMA Intern Med.

[9] Jabbarian LJ, Zwakman M, van der Heide A, Kars MC, Janssen DJ, van Delden JJ, Rietjens JA, Korfage IJ (2018). Advance care planning for patients with chronic respiratory diseases: a systematic review of preferences and practices. Thorax.

[10] Rhee JJ, Zwar NA, Kemp LA (2012). Uptake and implementation of advance care planning in Australia: findings of key informant interviews. Aust Health Rev.

[11] Thomas K, Ahmad N, Battye F (2013). Improving advance care planning and end of LIFE care in acute hospitals, using the gold STANDARDS framework acute hospital PROGRAMME. BMJ Support Palliat Care.

[12] Larson DG, Tobin DR (2000). End-of-life conversations: evolving practice and theory. JAMA.

[13] Winzelberg GS, Hanson LC, Tulsky JA (2005). Beyond autonomy: diversifying end-of-life decision-making approaches to serve patients and families. J Am Geriatr Soc.

[14] Harrington SE, Smith TJ (2008). The role of chemotherapy at the end of life:“when is enough, enough?”. JAMA.

[15] Evans N, Costantini M, Pasman H, Van den Block L, Donker GA, Miccinesi G, Bertolissi S, Gil M, Boffin N, Zurriaga O (2014). End-of-life communication: a retrospective survey of representative general practitioner networks in four countries. J Pain Symptom Manag.

[16] Lum HD, Sudore RL, Bekelman DB (2015). Advance care planning in the elderly. Medical Clinics.

[17] Kearley KE, Freeman GK, Heath A (2001). An exploration of the value of the personal doctor-patient relationship in general practice. Br J Gen Pract.

[18] Cursus Tijdig Spreken over het Levenseinde [https://www.nhg.org/scholing/nhg-verkorte-stip-cursus-tijdig-spreken-over-het-levenseinde-twee-modulen].

[19] Practice guides and tools [https://www.racgp.org.au/guidelines/advancecareplans].

[20] Respecting Choices Person-centered Care [http://www.learnrc.org/].

[21] Groot MM, Vernooij-Dassen MJ, Crul BJ, Grol RP (2005). General practitioners (GPs) and palliative care: perceived tasks and barriers in daily practice. Palliat Med.

[22] Groot MM, Vernooij-Dassen MJ, Verhagen SC, Crul BJ, Grol RP (2007). Obstacles to the delivery of primary palliative care as perceived by GPs. Palliat Med.

[23] De Vleminck A, Pardon K, Beernaert K, Deschepper R, Houttekier D, Van Audenhove C, Deliens L, Vander Stichele R (2014). Barriers to advance care planning in cancer, heart failure and dementia patients: a focus group study on general practitioners' views and experiences. PLoS One.

[24] Sharp Tim, Malyon Alexandra, Barclay Stephen (2017). GPs’ perceptions of advance care planning with frail and older people: a qualitative study. British Journal of General Practice.

[25] Boer T, Verkerk M, Bakker DJ: Over (−) behandelen, ethiek van de zorg voor kwetsbare ouderen. Ouderengeneeskunde 2014.

[26] Chilisa B, Kawulich B: Selecting a research approach: paradigm, methodology, and methods. C Wagner, B Kawulich, & M Garner, Doing social research: A global context 2012:51–61.

[27] Creswell JW, Poth CN (2017). Qualitative inquiry and research design: choosing among five approaches: sage publications.

[28] Boeije H (2002). A purposeful approach to the constant comparative method in the analysis of qualitative interviews. Qual Quant.

[29] O’Brien BC, Harris IB, Beckman TJ, Reed DA, Cook DA (2014). Standards for reporting qualitative research: a synthesis of recommendations. Acad Med.

[30] Unit TEI (2015). The 2015 Quality of Death Index. Ranking palliative care across the world. The Economist Intelligence Unit.

[31] De Vleminck A, Houttekier D, Pardon K, Deschepper R, Van Audenhove C, Vander Stichele R, Deliens L (2013). Barriers and facilitators for general practitioners to engage in advance care planning: a systematic review. Scand J Prim Health Care.

[32] Mitchell S, Loew J, Millington-Sanders C, Dale J (2016). Providing end-of-life care in general practice: findings of a national GP questionnaire survey. Br J Gen Pract.

[33] De Vleminck A, Batteauw D, Demeyere T, Pype P. Do non-terminally ill adults want to discuss the end of life with their family physician? An explorative mixed-method study on patients’ preferences and family physicians’ views in Belgium. Fam Pract. 2018;35(4):495–502. 10.1093/fampra/cmx125 29272418

[34] Claessen SJ, Francke AL, Engels Y, Deliens L (2013). How do GPs identify a need for palliative care in their patients? An interview study. BMC Fam pract.

[35] Dow LA, Matsuyama RK, Ramakrishnan V, Kuhn L, Lamont EB, Lyckholm L, Smith TJ (2010). Paradoxes in advance care planning: the complex relationship of oncology patients, their physicians, and advance medical directives. J Clin Oncol.

[36] Willard C, Luker K (2006). Challenges to end of life care in the acute hospital setting. Palliat Med.

[37] Thoonsen B, Groot M, Verhagen S, van Weel C, Vissers K, Engels Y (2016). Timely identification of palliative patients and anticipatory care planning by GPs: practical application of tools and a training programme. BMC palliative care.

[38] Murray SA, Kendall M, Grant E, Boyd K, Barclay S, Sheikh A (2007). Patterns of social, psychological, and spiritual decline toward the end of life in lung cancer and heart failure. J Pain Symptom Manag.

[39] Duenk R, Verhagen C, Bronkhorst E, van Mierlo P, Broeders M, Collard S, Dekhuijzen P, Vissers K, Heijdra Y, Engels Y (2017). Proactive palliative care for patients with COPD (PROLONG): a pragmatic cluster controlled trial. Int J Chron Obstruct Pulmon Dis.

[40] Mullick A, Martin J, Sallnow L (2013). An introduction to advance care planning in practice. BMJ.

[41] O'Neill O (2003). Some limits of informed consent. J Med Ethics.

[42] Novick G (2008). Is there a bias against telephone interviews in qualitative research?. Res Nurs Health.

[43] Drabble L, Trocki KF, Salcedo B, Walker PC, Korcha RA (2016). Conducting qualitative interviews by telephone: lessons learned from a study of alcohol use among sexual minority and heterosexual women. Qual Soc Work.

[44] Boyd Kirsty, Highet Gill, Crawford Debbie, Duthie Fiona, Murray Scott (2012). Identifying patients with advanced illness for needs assessment and care planning: SPICT in practice. BMJ Supportive & Palliative Care.

[45] Clayton JM, Hancock K, Parker S, Butow PN, Walder S, Carrick S, Currow D, Ghersi D, Glare P, Hagerty R (2008). Sustaining hope when communicating with terminally ill patients and their families: a systematic review. Psycho-Oncology.

[46] Olsman E, Leget C, Duggleby W, Willems D (2015). A singing choir: understanding the dynamics of hope, hopelessness, and despair in palliative care patients. A longitudinal qualitative study. Palliat Support Care.

[47] Koenig Kellas J, Castle KM, Johnson A, Cohen MZ (2017). Communicatively constructing the bright and dark sides of hope: family caregivers’ experiences during end of life cancer care. Behavioral Sciences.

[48] Siouta N, Van Beek K, Van der Eerden M, Preston N, Hasselaar J, Hughes S, Garralda E, Centeno C, Csikos A, Groot M (2016). Integrated palliative care in Europe: a qualitative systematic literature review of empirically-tested models in cancer and chronic disease. BMC palliative care.

[49] Reeves E, Liebig B, Schweighoffer R (2018). An investigation of barriers and facilitators of coordination between primary palliative care and specialized palliative care in Switzerland: a qualitative study. Zeitschrift für Palliativmedizin.

[50] Petrova Mila, Riley Julia, Abel Julian, Barclay Stephen (2016). Crash course in EPaCCS (Electronic Palliative Care Coordination Systems): 8 years of successes and failures in patient data sharing to learn from. BMJ Supportive & Palliative Care.

[51] Verhaegh KJ, MacNeil-Vroomen JL, Eslami S, Geerlings SE, de Rooij SE, Buurman BM (2014). Transitional care interventions prevent hospital readmissions for adults with chronic illnesses. Health Aff (Millwood).

[52] Buurman BM, Parlevliet JL, Allore HG, Blok W, van Deelen BA, van Charante EPM, de Haan RJ, de Rooij SE (2016). Comprehensive geriatric assessment and transitional care in acutely hospitalized patients: the transitional care bridge randomized clinical trial. JAMA Intern Med.

[53] Van Wijlick E, Van Dieten E, Wigersma L. Elf spelregels voor praten over het einde. Medisch Contact. 1968:33–4.

[54] Keirse M, Vlaanderen OFPZ: Het levenseinde teruggeven aan de mensen. Over vroegtijdige planning van de zorg Federatie Palliatieve Zorgen: Wemmel Downloadbare brochure op www palliatief be 2009.

[55] Lynn J, Adamson DM: Living well at the end of life Adapting health care to serious chronic illness in old age. In*.*: DTIC Document; 2003.

[56] Molewijk A, Abma T, Stolper M, Widdershoven G (2008). Teaching ethics in the clinic. The theory and practice of moral case deliberation. J Med Ethics.

