# Supplementary material for: Advance care planning conversations with palliative patients: looking through the GP’s eyes
Source: BMC Fam Pract. 2018 Nov 28;19:184. doi: 10.1186/s12875-018-0868-5 (PMC6263059; doi:10.1186/s12875-018-0868-5)
Supplement: Supplementary file 1 — Content ACP training (NHG) [53–56]. (DOCX 18 kb) [file 12875_2018_868_MOESM1_ESM.docx]

Additional file 1. Content ACP training (NHG)

| Module I.  The learning goals in this module were partly theoretical, partly practical.  *Theoretical*   - An introduction to ACP - ‘Rules’ when talking about the end of life, based on theory from “Medisch Contact”, a Dutch magazine for physicians.[53] - Roadmap for early discussing the care plan, based on the theory of Belgian psychologist Manu Keirse.[54]   *Practical*   - Discussion about by GP’s prepared case, for which homework was done.   *Subsequently*   - Discussing premeditated questions based on a – by the trainers introduced – exercise case. - In that discussion, theory was nested about;   *… the moment of starting palliative care; model Lynn en Adamson.[55] … different types of living wills.  … discussing a case by means of Moral Case Deliberation.[56] … different types of patients and cultures in ACP.* |
| --- |
| Module II.  Module II had a more *interactive* content.   - GPs worked in small groups; - Brought in a difficult care from practice (in between the modules the homework assignment was to start an ACP discussion with three patients); - Re-enacted this case by means of role playing – one GP played GP, one played patient, one was observer; - After the role play, GPs gave each other feedback, and discussed what they thought was went well, and what was difficult; - In this way, GPs shared ‘tips and tricks’ and reflected on each others’ action and attitudes; - Finally, the sessions were discussed plenary. |
