# Supplementary material for: Advance care planning conversations with palliative patients: looking through the GP’s eyes
Source: BMC Fam Pract. 2018 Nov 28;19:184. doi: 10.1186/s12875-018-0868-5 (PMC6263059; doi:10.1186/s12875-018-0868-5)
Supplement: Supplementary file 2 — Interview guide. (DOCX 16 kb) [file 12875_2018_868_MOESM2_ESM.docx]

Additional file 2. Interview guide

| **Main question:** *“How do GPs who participated in the NHG training “Timely discussing EoL issues” experience the application of ACP with palliative patients?”*  Background, motivation and experiences with EoLC   - Why did you sign up for the training and what are your experiences with EoL care? - What is the relevance of ACP in palliative patients? What significance/meaning does ACP has for you? - ACP is on the medical and policy agenda more and more, do you notice that in practice, or is it of all times? - How do you perceive your role as a GP in proactively discussing the EoL?   Starting the discussion   - After the training, you actively started ACP discussions with palliative patients. Can you give an example of how such a discussion takes place? - How do you feel about timely starting the discussion? - When does ACP work? When is it difficult?   - How do patients and relative experience it? Why do you think that is?   - According to you, what do patients need the most when starting ACP? - Does ACP also have its drawbacks, and if so; what are they?   Ethical aspects of implementing ACP in practice implementation ACP in practice   - What do you come across when you introduce ACP with patients and relatives? - What role do the principles of ‘the duty to inform’ and ‘the right not to know’ play for you in ACP? - ACP is about QoL, which also covers not over treating patients. Do you have examples in which this went well / wrong? - If – according to you – patients are curatively over treated, do you discuss this with the specialist concerned? - Do the expediency and usefulness of potential future treatments play a role for you? |
| --- |
